# Supplementary material for: Evolution of DNA methylation in the human brain
Source: Nat Commun. 2021 Apr 1;12:2021. doi: 10.1038/s41467-021-21917-7 (PMC8017017; doi:10.1038/s41467-021-21917-7)

Supplementary Information

Evolution of DNA methylation in the human brain

Jeong, Mendizabal et al.

Supplementary Fig. 1. Overview of the workflow for WGBS and WGS data processing and differential methylation analyses.

	Step	Step Description
1	**WGBS library preparation**	DNA extraction<br>Bisulfite conversion of DNA<br>Library preparation and QC
2	**Sequencing**<br>(Hi-SeqX)	Paired-end Illumina sequencing
3	**Adapter and quality trimming**<br>(Trim Galore v.0.4.1, FastQC)	Trim low quality base-pairs at read ends<br>Trim adapters sequenced within reads<br>Evaluate read quality
4	**PhiX removal**<br>(Bowtie2 v2.2.7)	Removal of phiX spike-in control
5	**Lambda mapping**<br>(Bowtie2 v2.2.7)	Estimate bisulfite conversion rates
5	**Mapping**<br>(Bowtie2 v2.2.7, Bismark v0.19.0)	Map reads to respective genome
6	**Deduplication**<br>Bismark v0.19.0)	Remove potential PCR duplicates
7	**Methylation extraction**<br>(Bismark v0.19.0)	Estimate per base fractional methylation<br>mCpg/CpG
8	**Differential Methylation Analysis**	

	Step	Step Description
1	**WGS library preparation**	DNA extraction<br>Library preparation and QC
2	**Sequencing**<br>(Hi-SeqX)	Paired-end Illumina sequencing
3	**Adapter and quality trimming**<br>(Trim Galore v.0.4.1, FastQC)	Trim low quality base-pairs at read ends<br>Trim adapters sequenced within reads<br>Evaluate read quality
4	**Mapping**<br>(bwa v 0.7.4-r385)	Map reads to respective genome
5	**Deduplication**	Remove potential PCR duplicates
6	**Variant Discovery**	SNP calling

Exclusion of polymorphic CpG sites (C->T and G->A)

Supplementary Fig. 2. Comparison of evolutionary conservation scores demonstrate rapid divergence of CG contexts. We used PhastCons and PhyloP for evolutionary conservation scores. Specifically, we classified all cytosines into four different cytosine contexts based on the subsequent base (i.e. CA, CC, CG, and CT). For each cytosine context, we computed a mean conservation score of 100,000 randomly selected cytosine sites. Then, we repeated this process 1,000 times and compared the mean conservation scores across cytosine contexts. The Y-axis represents the mean conservation score resulting from the cytosine of 100,000 randomly selected positions in each context. Each boxplot displays the mean conservation scores of 1,000 trials. Box represents a range from the first quartile to the third quartile. The line in the box indicates the median value. The minima and maxima are within 1.5 times the distance between the first and third quartiles from box.

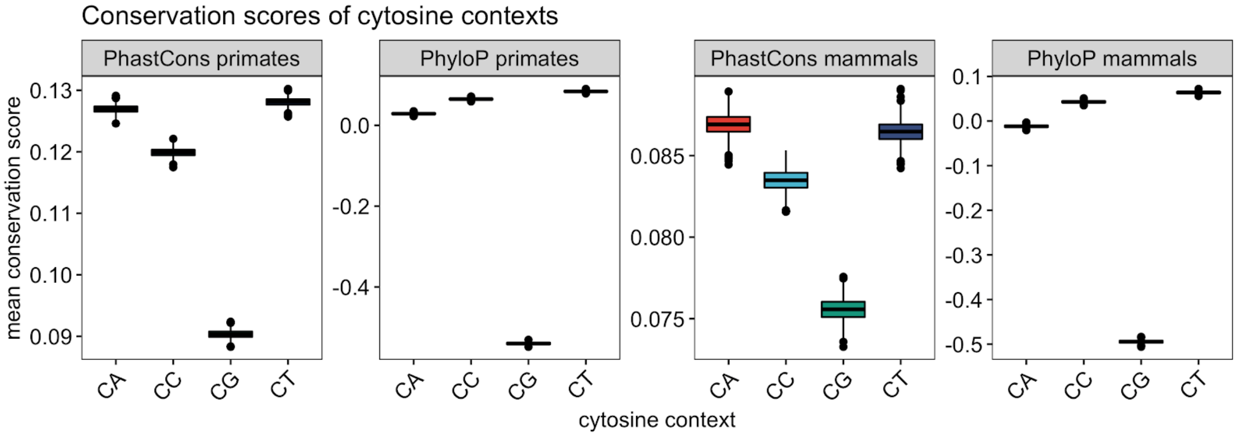

Supplementary Fig. 3. Loss of CG sites over evolutionary time. **(A)** Comparison of the total number of CpGs in either the human (hg19) or chimpanzee (panTro5) genomes with the total number of conserved CpGs in genic regions in both genomes or in non-genic regions in both genomes. (**B)** Comparison of the total number of CpGs in human (hg19) or macaque (rheMac8) genomes with the total number of conserved CpGs in genic regions in both genomes or in non-genic regions in both genomes. **(C)** Dinucleotide composition of CpGs of rheMac8 in the hg19 genome.

A

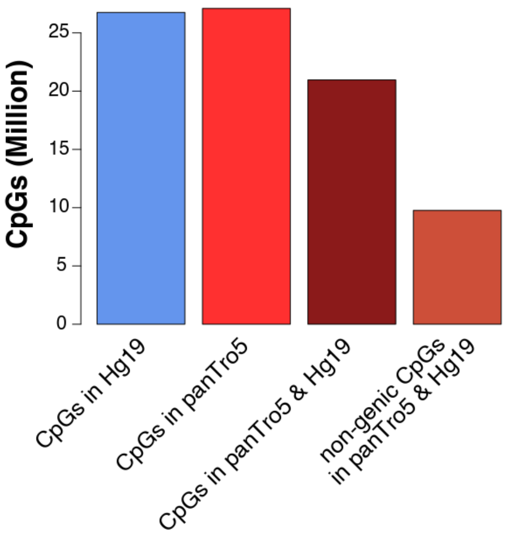

B

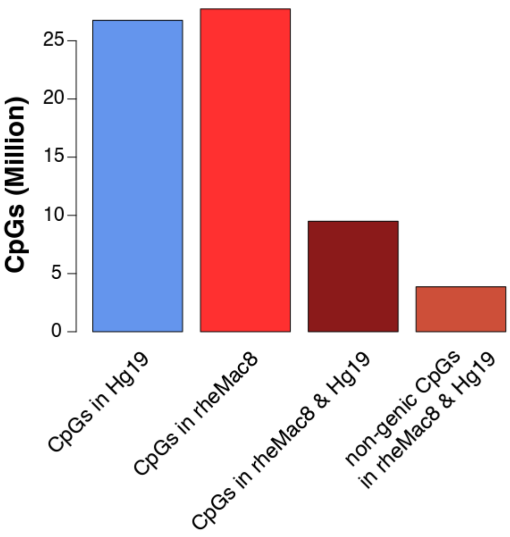

C

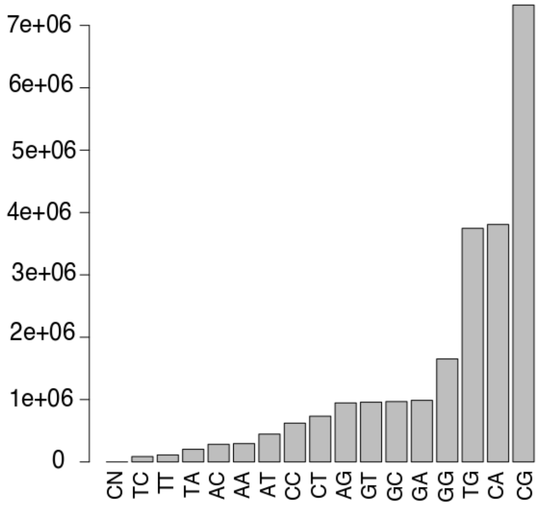

Supplementary Fig. 4. Comparison of fractional methylation between conserved CG sites and species-specific CG sites in humans. To illustrate, a subset of 100,000 sites were selected for each CG group. A one-tailed t-test was conducted to test whether conserved CG sites are biased toward hypomethylation. Box represents a range from the first quartile to the third quartile. The line in the box indicates the median value. The minima and maxima are within 1.5 times the distance between the first and third quartiles from box.

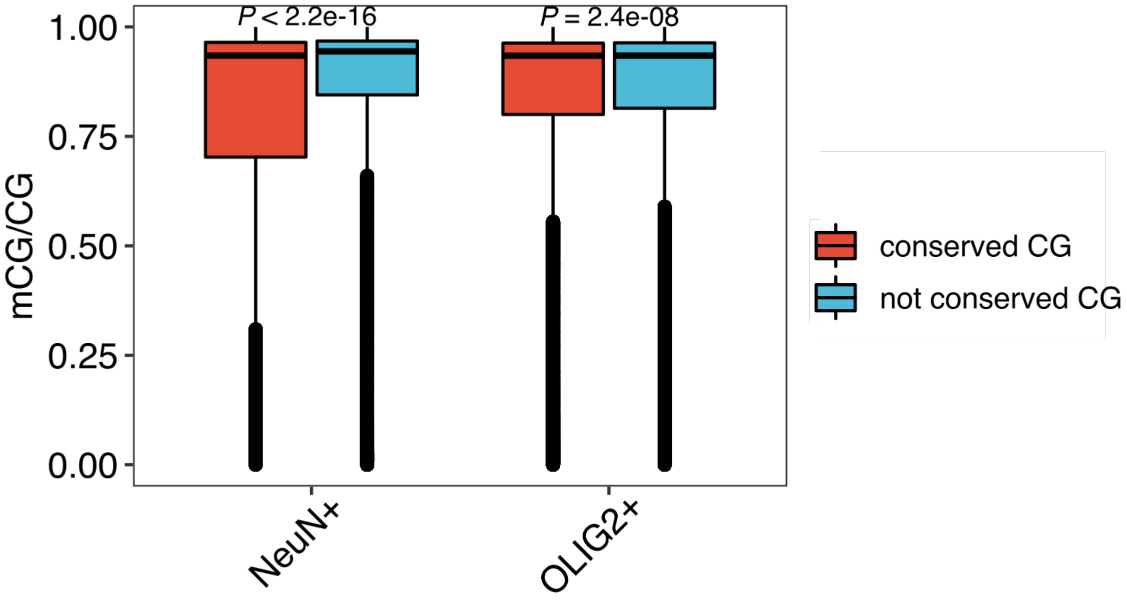

Supplementary Fig. 5. Distribution of evolutionarily conserved CpGs and variable CpGs in different functional genomic regions. Fold-enrichment was computed from the occurrences of the CpGs for each feature compared to random control sets (n=100). Red dashed lines indicate fold-enrichment values of 1.

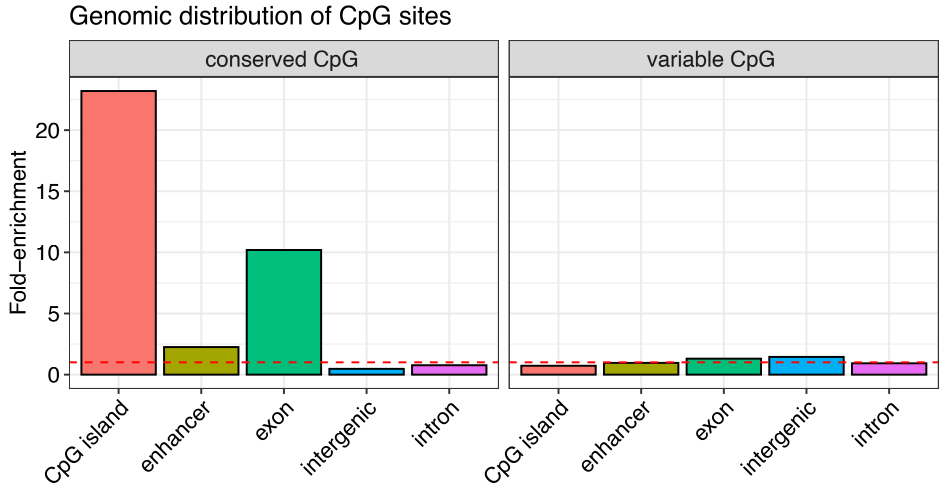

Supplementary Fig. 6. Transcription factor families with differential motif enrichment in conserved CpGs (gray dots) vs. human-specific-CpGs (variable CpGs, blue dots) in (A) core v11 and (B) full HOCOMOCO v11 databases.

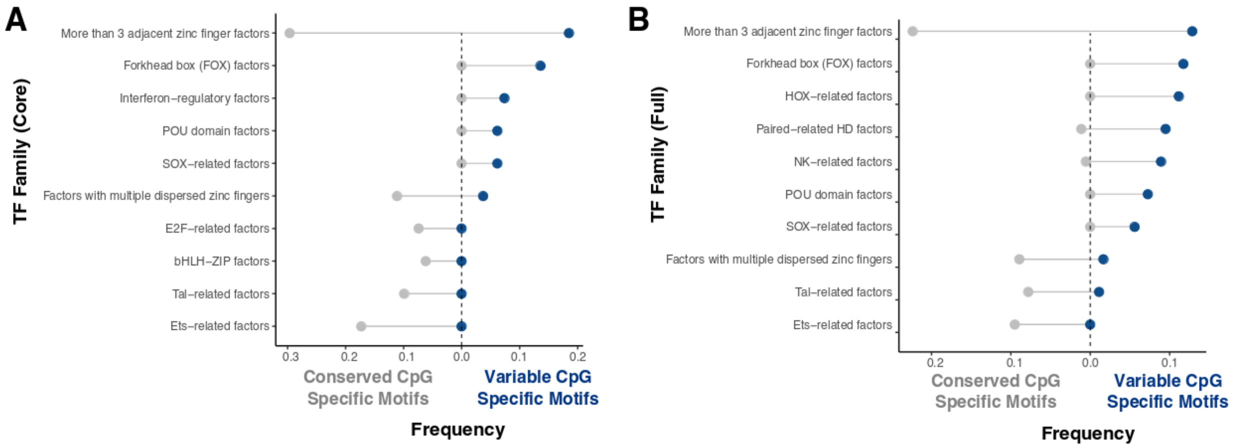

Supplementary Fig. 7. Hierarchical clustering of CG methylomes for **(A)** Chimpanzees and **(B)** macaques. Names ending in ND represent neuronal (NeuN+) cell samples and names ending in OD represent oligodendrocyte (OLIG2+) cell samples. (**C**) An example CG DMR between NeuN+ and OLIG2+ cells spanning the entirety of *QKI*, a gene involved in oligodendrocyte function. This gene is consistently hypomethylated in oligodendrocytes compared to neurons in all three species.

A

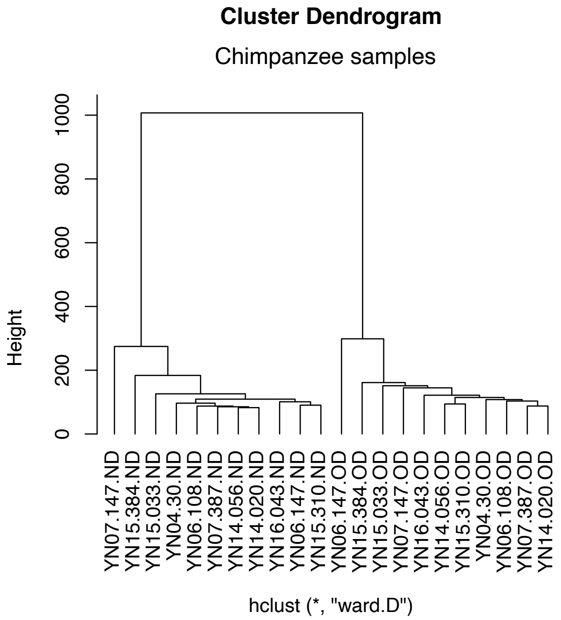

B

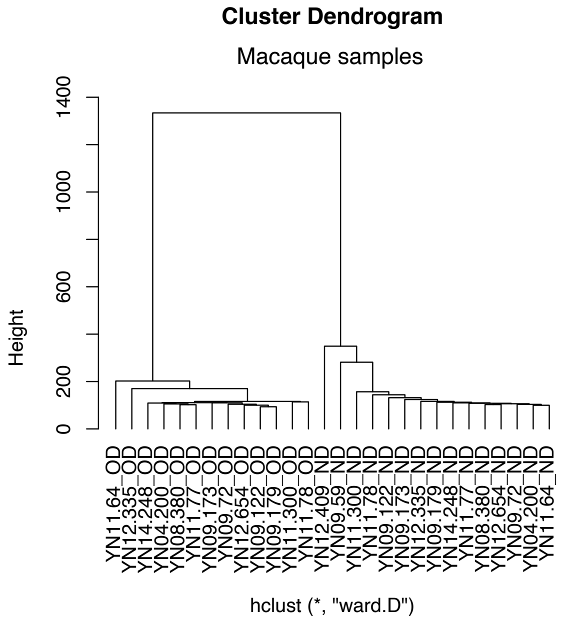

C

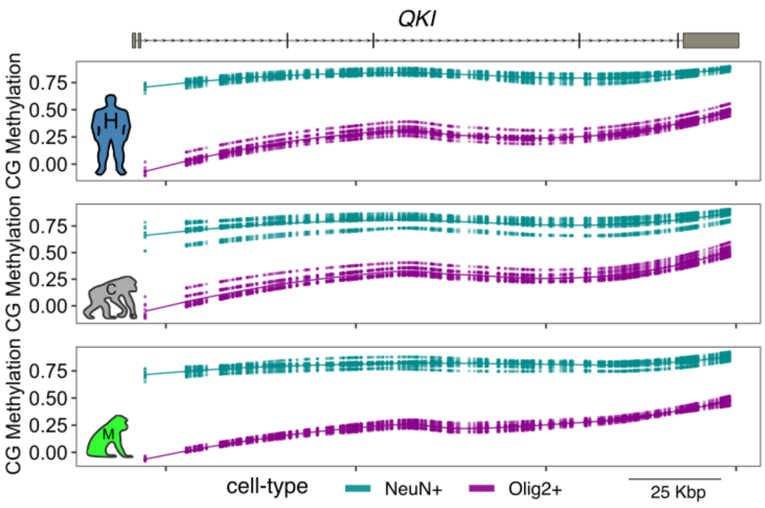

Supplementary Fig. 8. (A) Genomic locations of human DMRs show that most DMRs are within or nearby genes and **(B)** enriched in promoters and genic regions. Fold enrichment is computed by occurrences of DMRs in each genomic feature compared to GC matched control region sets (n=100).

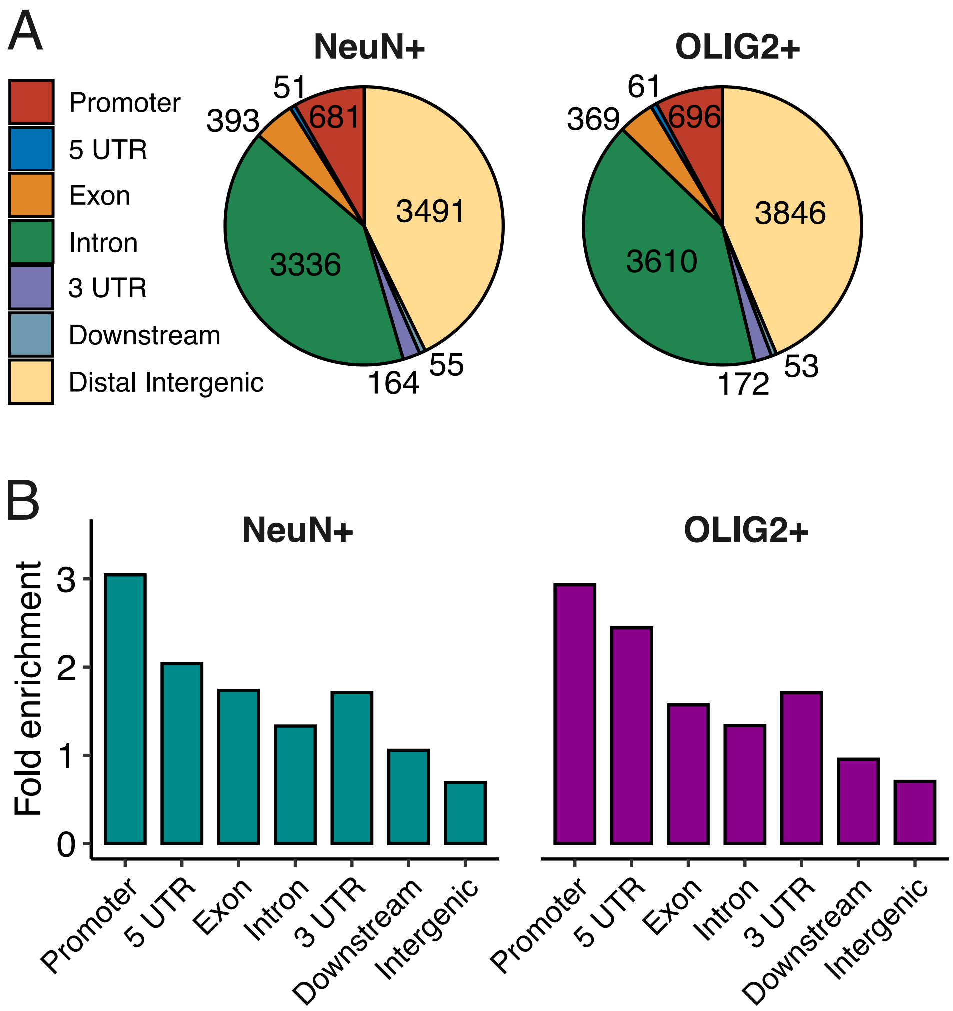

Supplementary Fig. 9. Genomic windows (200bps each) containing mQTLs are more often associated with DMRs than genomic windows containing SNPs matched for their minor allele frequency (MAF) (control) of the same size across different GC contents.

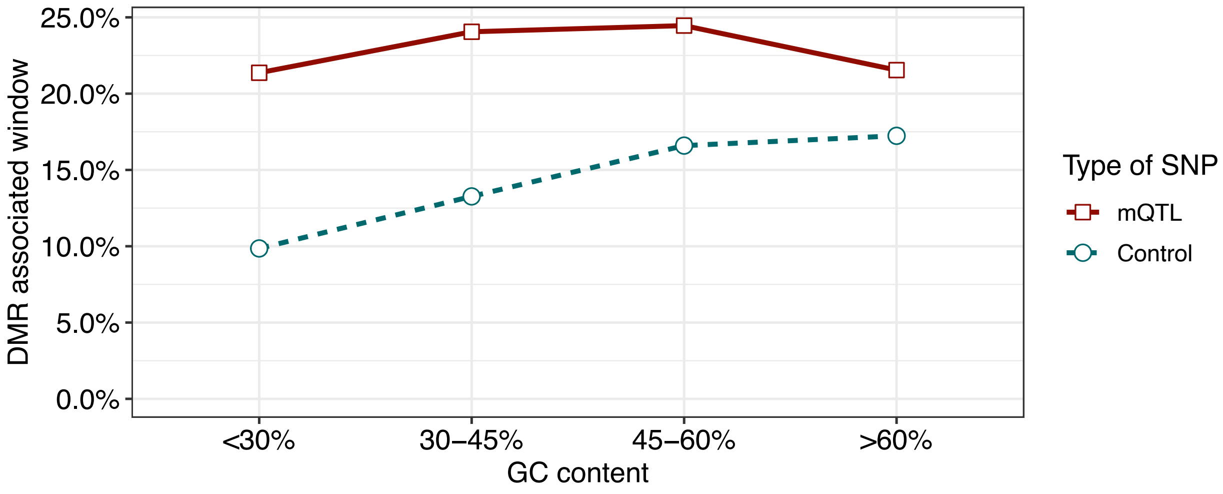

Supplementary Fig. 10. Genomic locations of human neuron-specific hypomethylated CG DMRs are enriched in cell-type specific human brain epigenetic marks. Fold enrichment is computed by observed numbers of DMRs (red dashed line) overlapping with each neuron-specific epigenetic mark compared to GC matched control region sets (n=100).

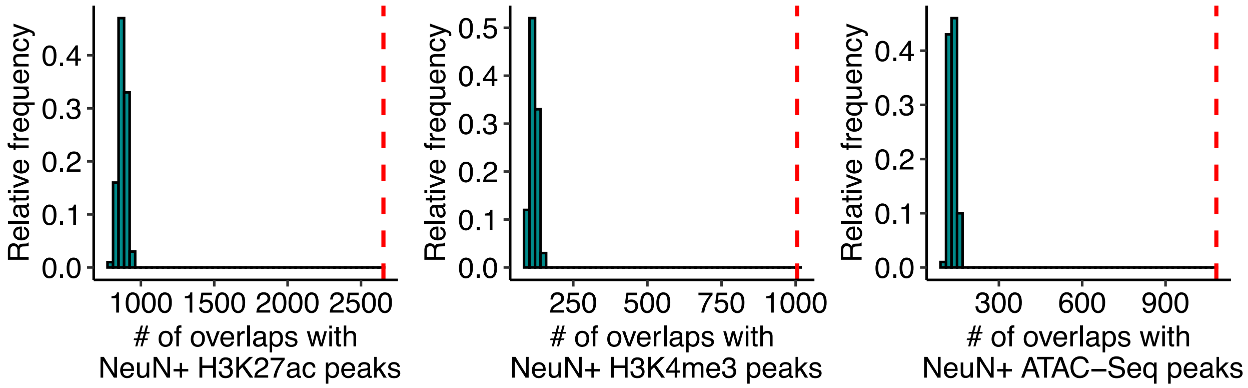

Supplementary Fig. 11. Enhancer enrichment at human-specific hypomethylated CG DMRs. 25 chromatin state-model maps based on 6 chromatin mark ChIP-Seq experiments (H3K4me3, H3K4me1, H3K36me3, H3K27me3, H3K9me3 and H3K27ac) were obtained from the Roadmap Epigenomics Project. Each dot represents the enrichment for enhancer-related states (TxReg, TxEnh5', TxEnh3', TxEnhW, EnhA1, EnhA2, EnhW1, EnhW2, and EnhAc) compared to 100 sets of GC-content matched control DMR sets for a given cell-type or tissue. The original 117 cell-type and tissue-types were grouped into 11 categories shown in the y-axes (total number of cell-type/tissues per group is indicated). Empirical *P*<0.01 for all enrichments.

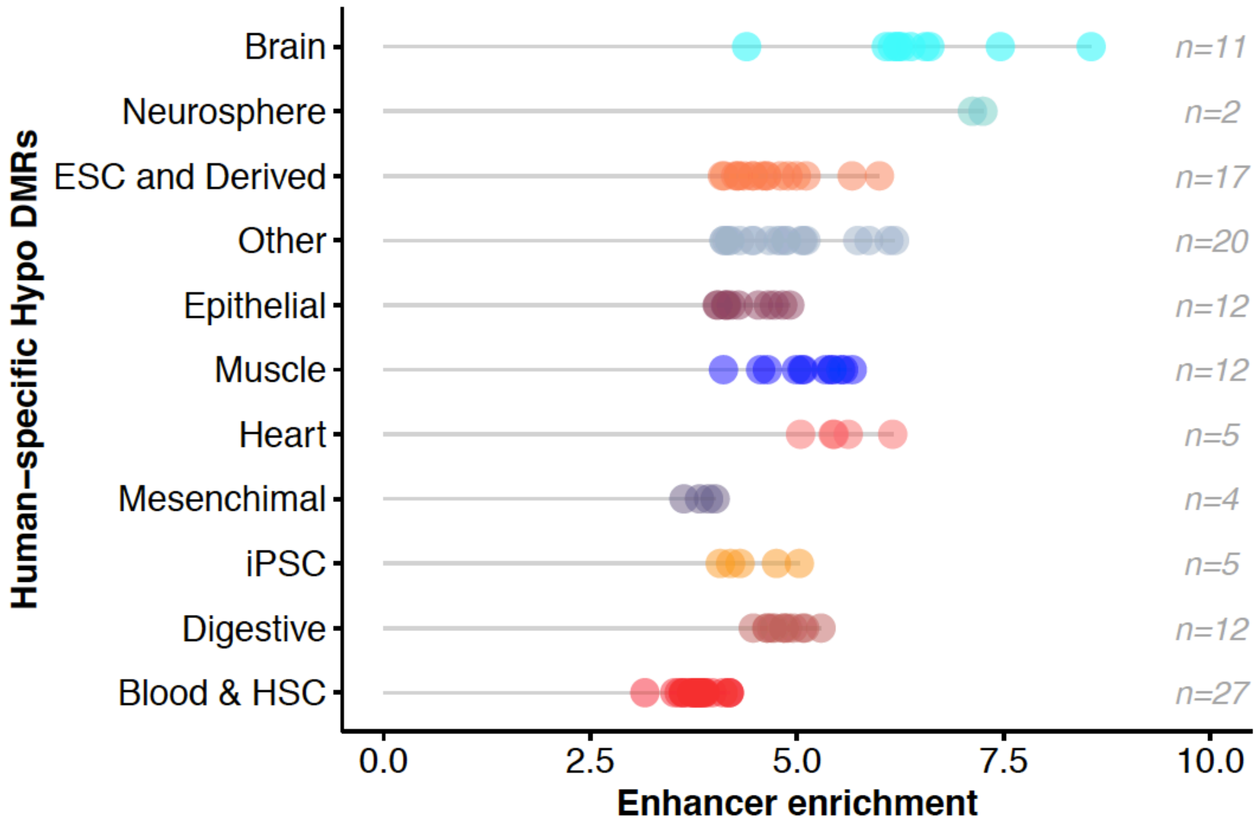

Supplementary Fig. 12. (A) Human neuron-hypo CG DMRs are significantly co-localized with enhancer-promoter (E-P) pairs and ncHARs (red dashed lines). Null distributions were plotted based on the GC matched control region sets (n=100) that overlap with enhancer-promoter pairs and ncHARs. **(B)** Chimpanzee DMRs that overlap with enhancer-promoter pairs and ncHARs were falling in the distribution based on the GC matched control region sets (n=100) that overlap with enhancer-promoter pairs and ncHARs.

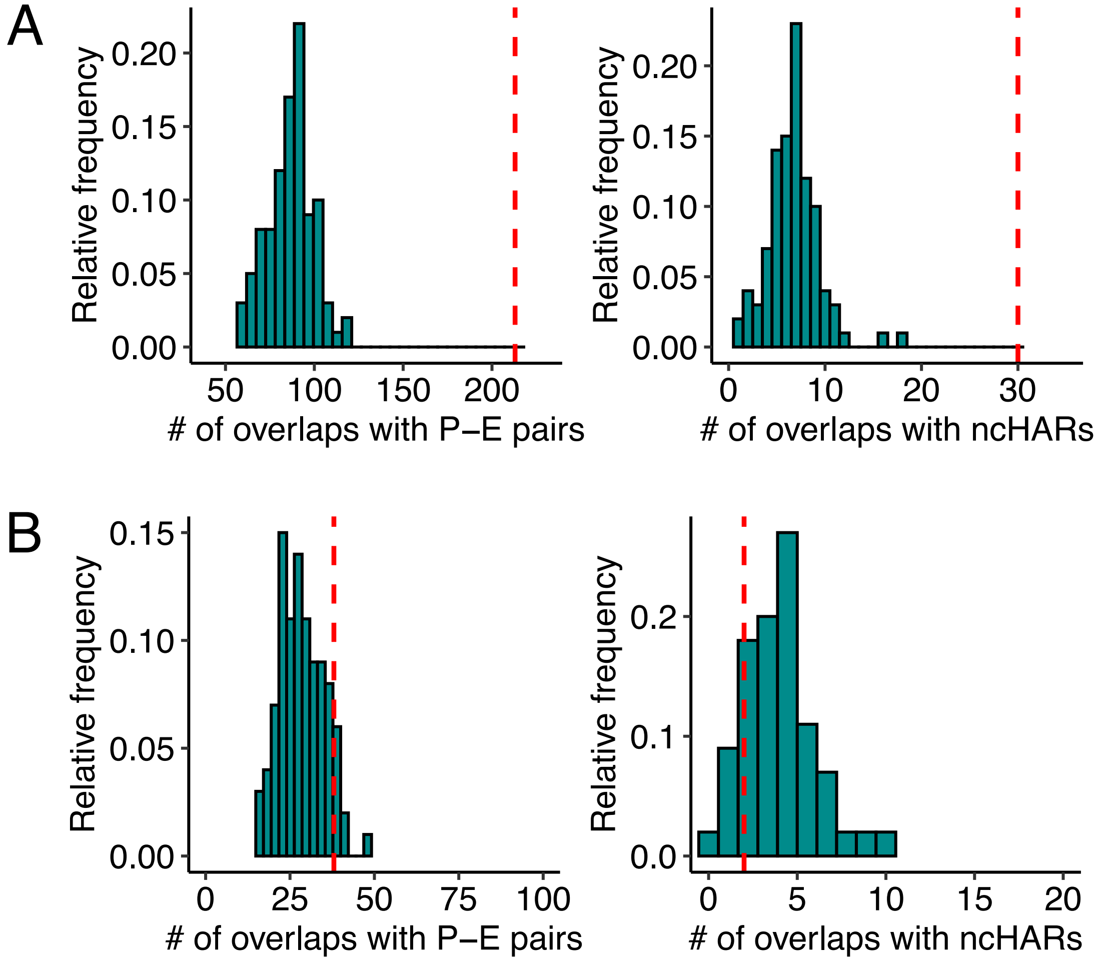

Supplementary Fig. 13. (A) Distribution of DNA methylation differences between human and chimpanzee brain cell-types at human-specific hypomethylated DMRs with and without enriched TF motifs. P-values for one-sided Wilcoxon signed-ranked test with alternative = greater (TF n=2439 vs non-TF n=652 for each cell-type). (B) Same as in panel A but separated by the specific TF enriched (*FOXP1* n=1996, *FOXK1* n=1906, *NFIC* n=462 and non-TF n=652 for each cell-type). (C) Distribution of gene expression differences between human and chimpanzee at human-specific hypomethylated DMRs with and without enriched TF motifs. P-values for one-sided Wilcoxon signed-ranked test with alternative = greater (NeuN+: TF n=1110 vs non-TF n=7262, and OLIG2+: TF n=1031 vs non-TF n=6529). (D) Same as in panel C but separated by the specific TF enriched (NeuN: *FOXP1* n=957, *FOXK1* n=901, *NFIC* n=248, non-TF n=7262, and OLIG2: *FOXP1* n=892, *FOXK1* n=833, *NFIC* n=229 and non-TF n=6529). Box represents a range from the first quartile to the third quartile. The line in the box indicates the median value. The minima and maxima are within 1.5 times the distance between the first and third quartiles from box.

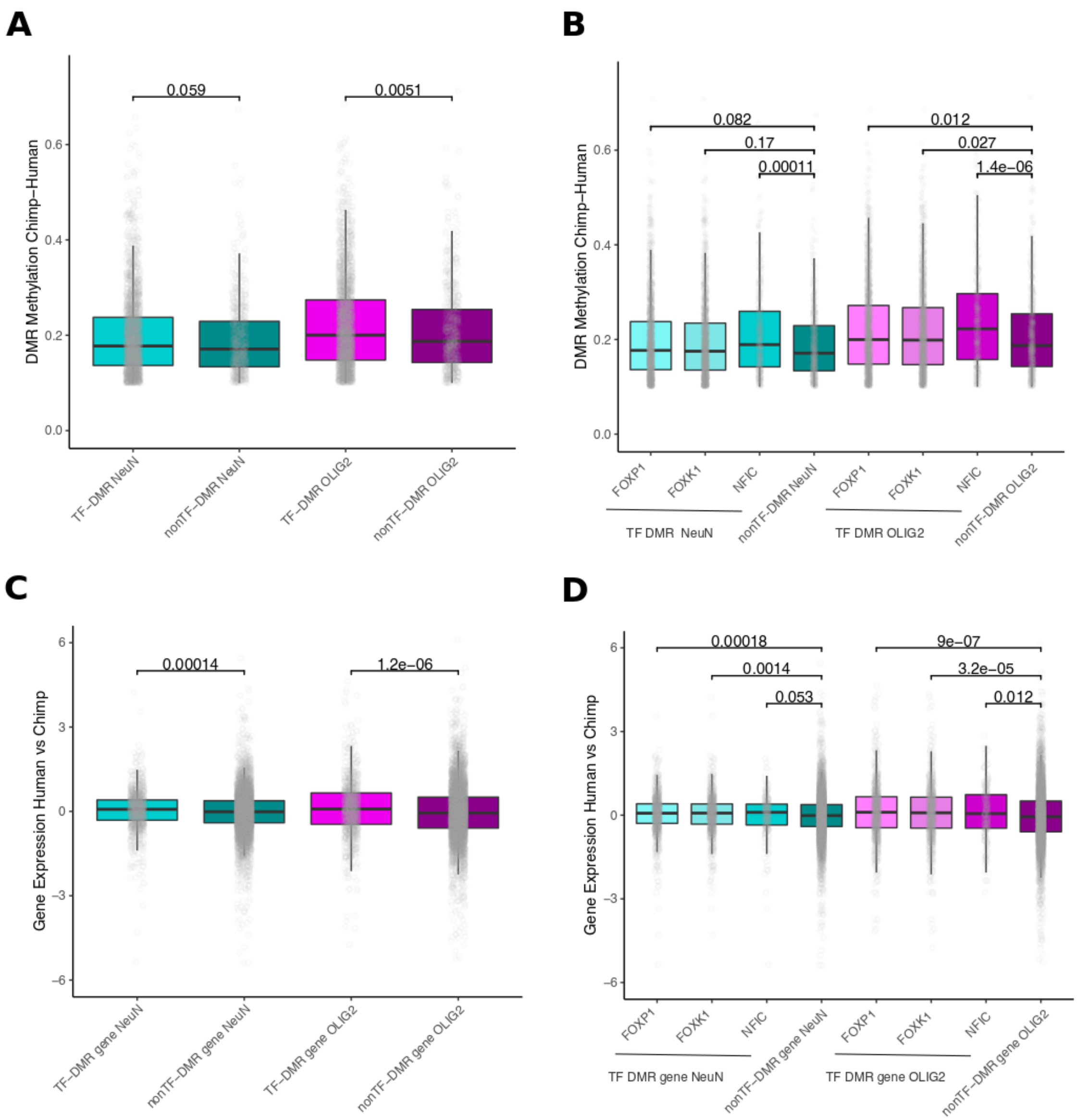

Supplementary Fig. 14. Evolutionary lineages and their divergence time among the three primate species. Human-specific methylation changes are depicted in blue and chimpanzee-specific methylation changes are shown in grey. Sites in which macaque show methylation divergence from human and chimpanzee but exhibit no difference between human and chimpanzee are denoted in green. Numbers of CH sites showing significant methylation changes on the three evolutionary lineages are plotted.

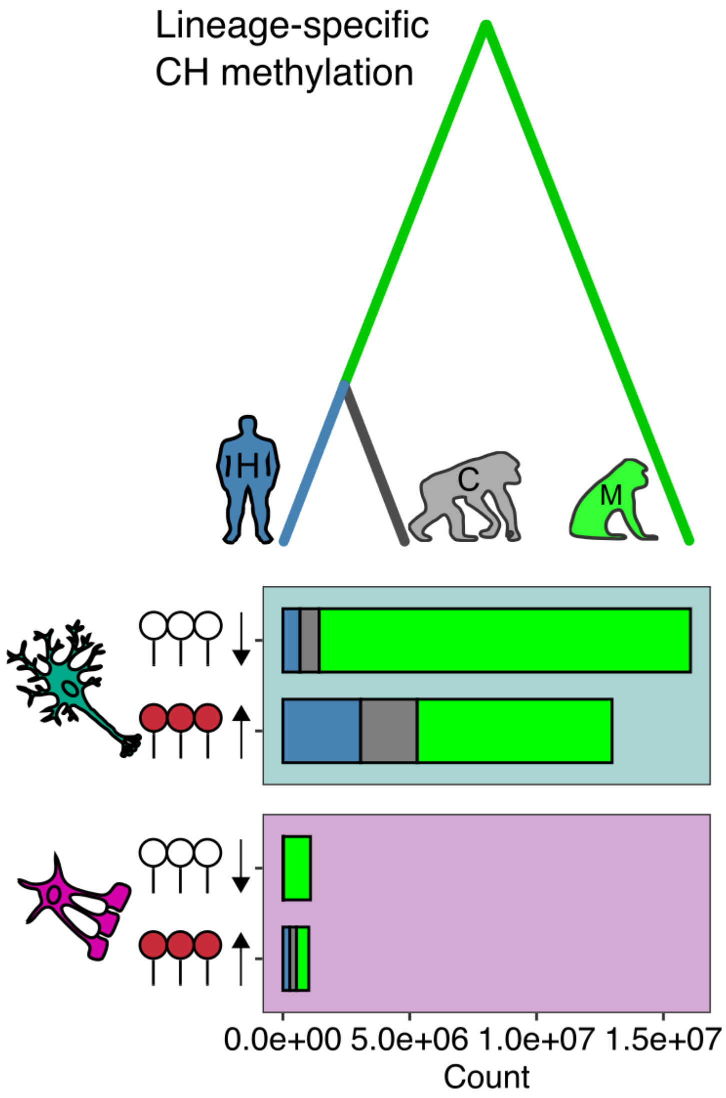

Supplementary Fig. 15. Correlation between gene expression and CH methylation in neuronal cells across all three primate species.

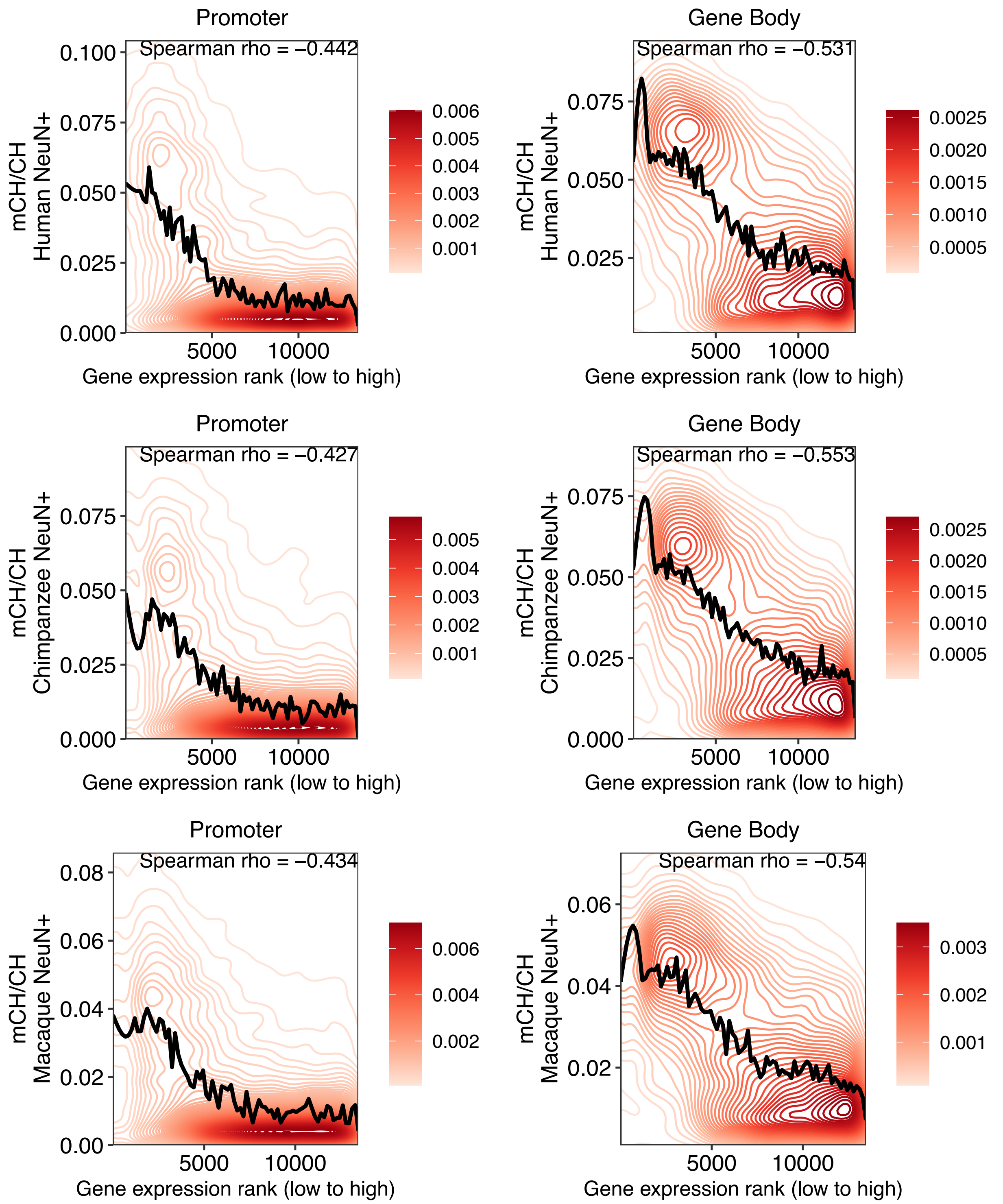

Supplementary Fig. 16. Correlation coefficient between different methylation contexts and between methylation and gene expression in human NeuN+.

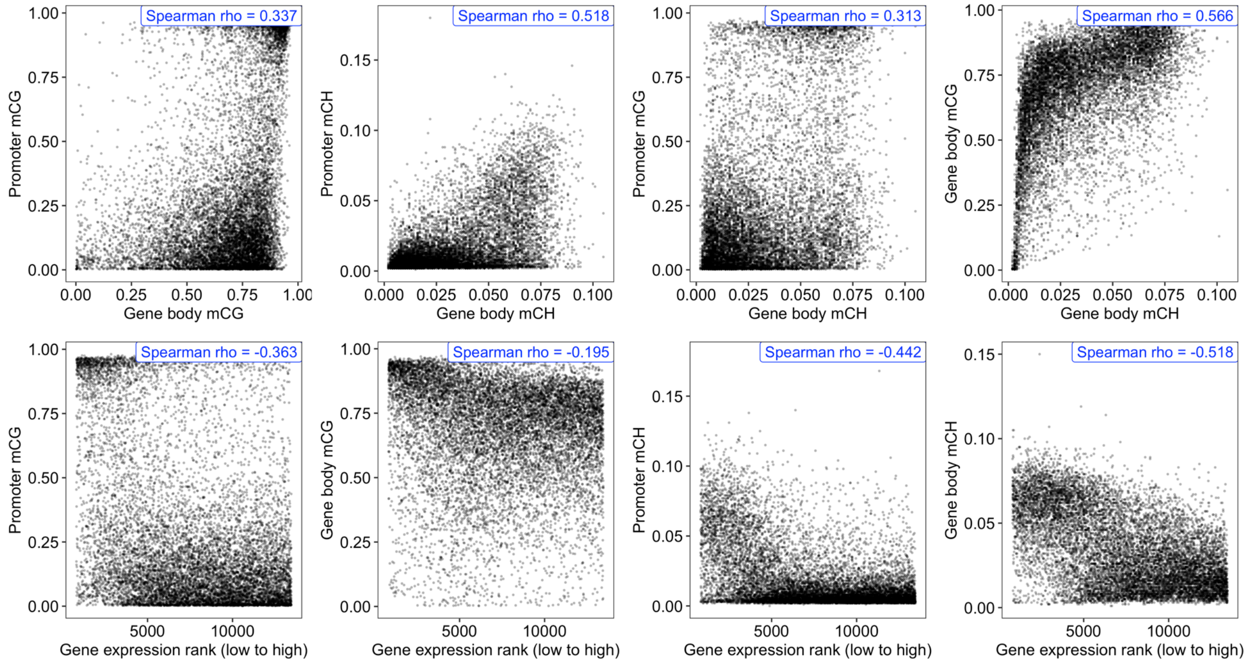

Supplementary Fig. 17. Relationship between CH gene body methylation (relative difference between species, X-axis) and CG promoter methylation (relative difference between species, Y-axis) in different gene types. DEG: differentially expressed gene list from Berto et al. 2019.

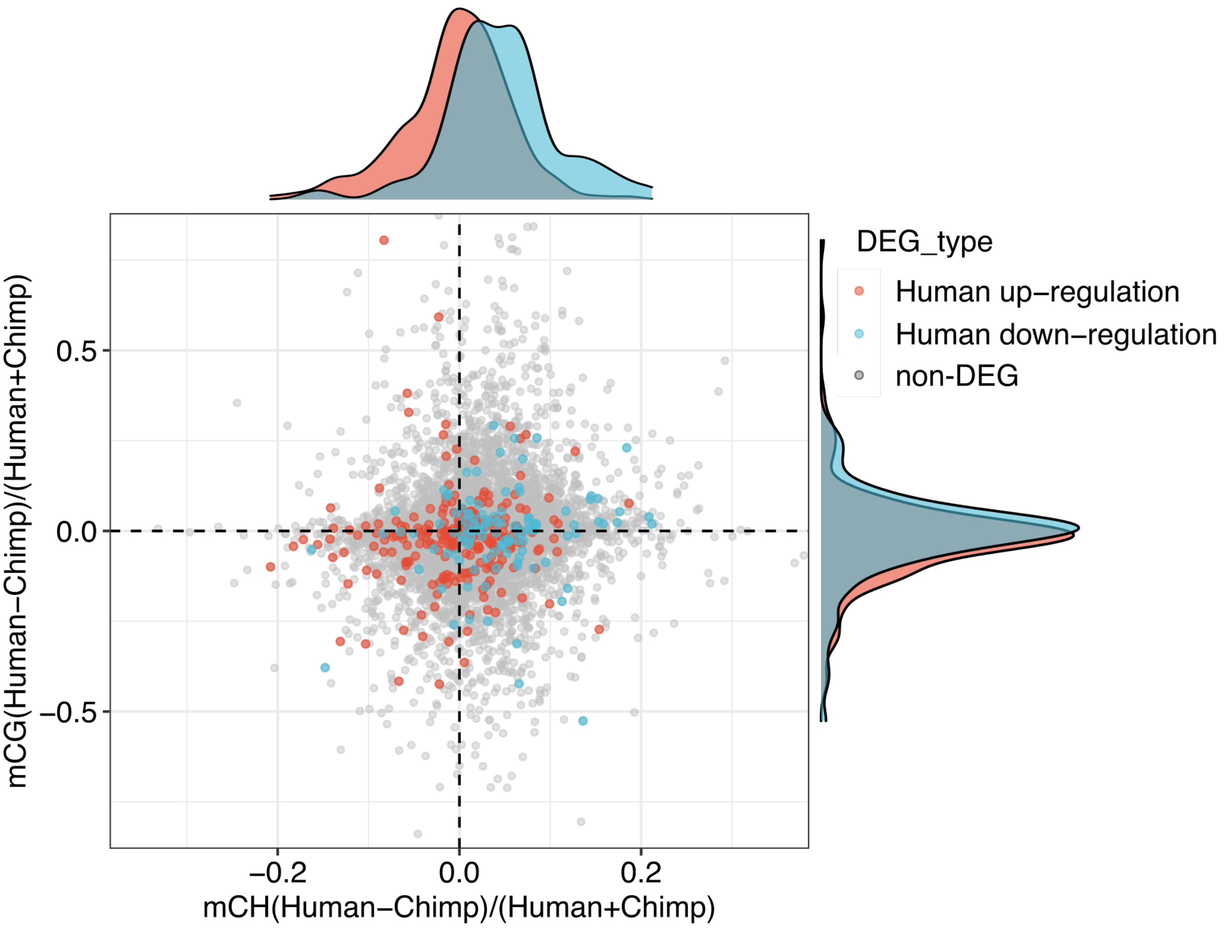

Supplementary Fig. 18. (A) Gene expression in human and macaque for CH DMR genes over developmental time points (Human CH DMR genes, *n* = 450 and Chimpanzee CH DMR genes, *n* =144). Macaque samples were age-matched to human developmental time points (PCW: Post conception week; M: Month; Yr: Year). Same as in panel A but (B) using CG DMR genes (hypo; Human DMR genes, *n* = 742 and Chimpanzee DMR genes, *n* = 201) and (C) using CG DMR genes (hyper; Human DMR genes, *n* = 223 and Chimpanzee DMR genes, *n* = 627). Box represents a range from the first quartile to the third quartile. The line in the box indicates the median value. The minima and maxima are within 1.5 times the distance between the first and third quartiles from box.

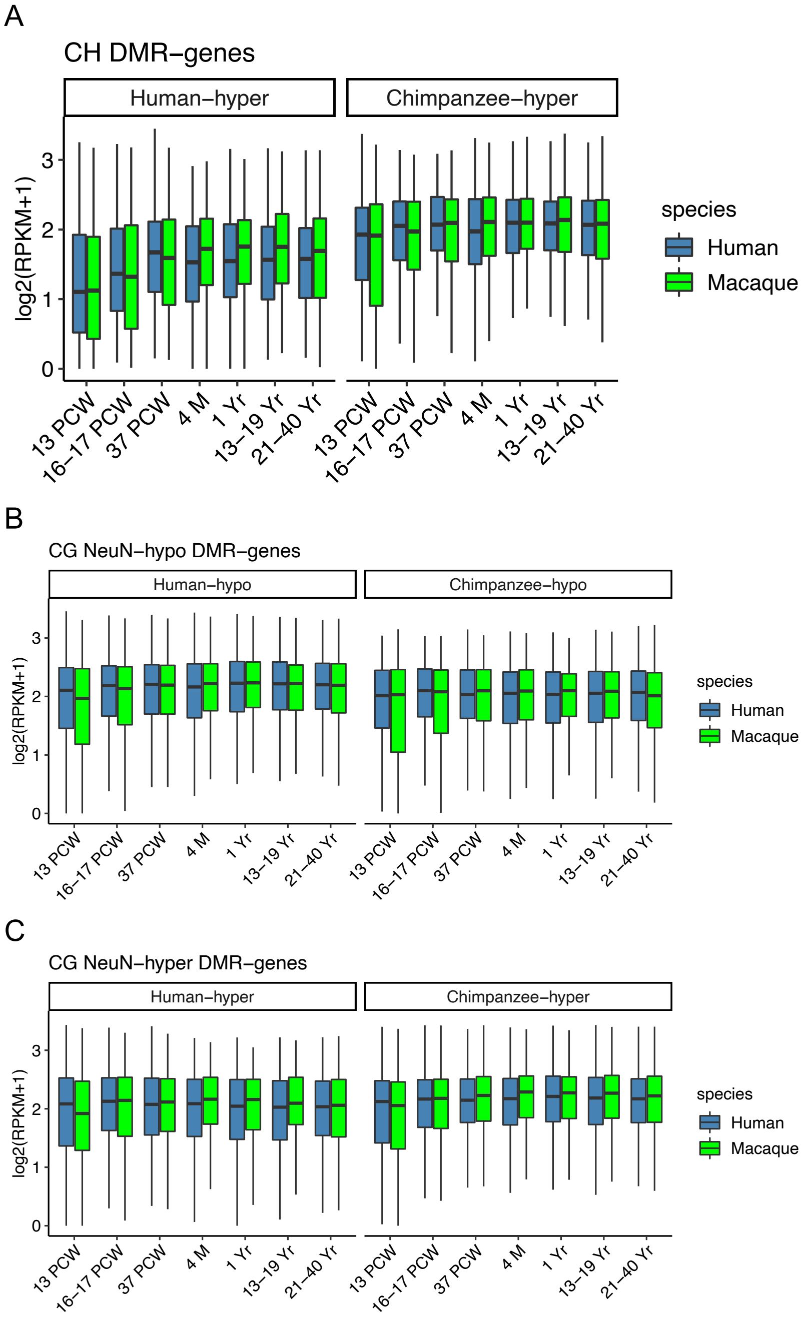

Supplementary Fig. 19. Gene expression of lineage-specific CH DMR genes (Human CH DMR genes, *n* = 590 and Chimpanzee CH DMR genes, *n* =159) in neuronal (NeuN+) cell samples and oligodendrocyte (OLIG2+) cell samples. Statistical significance was calculated using two-sided Mann-Whitney U-test. Box represents a range from the first quartile to the third quartile. The line in the box indicates the median value. The minima and maxima are within 1.5 times the distance between the first and third quartiles from box.

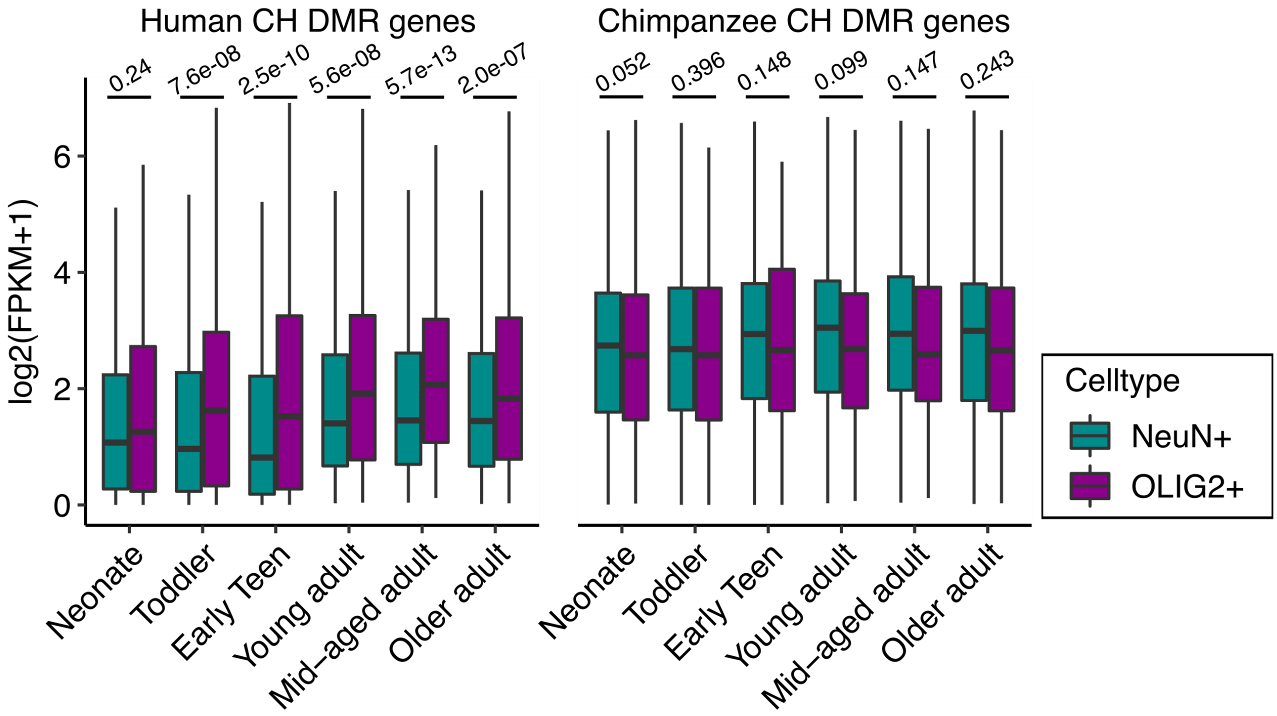

Supplementary Fig. 20. CH methylation of single cell methylomes. **(A)** Boxplots display average methylation differences between human CH DMR genes (*n* = 674) and Chimpanzee CH DMR genes (*n* = 173) of neuronal cells for neuronal subtypes. **(B)** As a control, we randomly selected the same number of genes. Box represents a range from the first quartile to the third quartile. The line in the box indicates the median value. The minima and maxima are within 1.5 times the distance between the first and third quartiles from box.

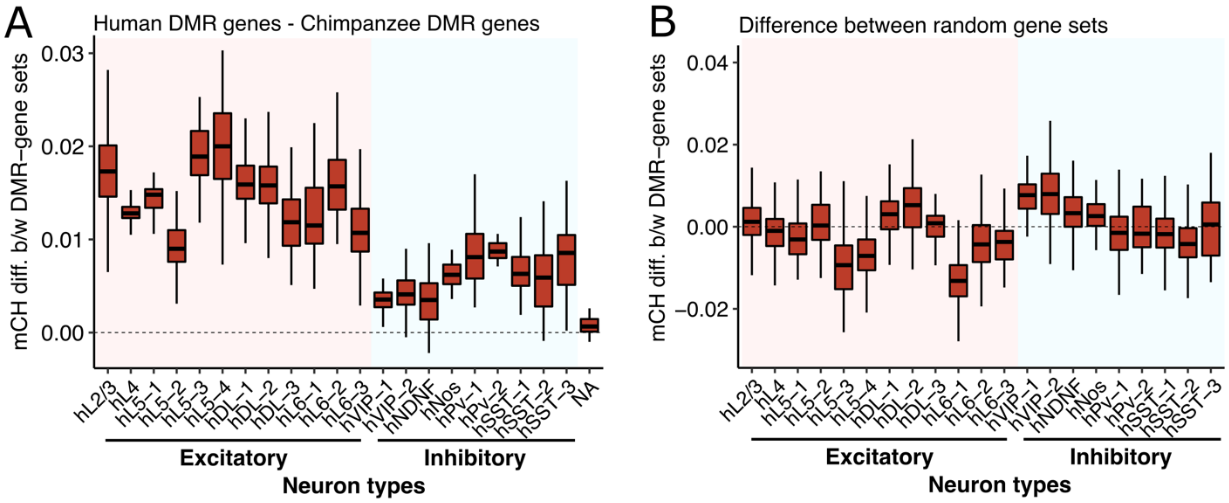

Supplementary Fig. 21. **(A) and (B)** Enrichment and P-values for schizophrenia heritability at 100 subsets of conserved NeuN+ Hypo DMRs that match the number and length of human-derived NeuN+ Hypo DMRs. Boxplots show the results of 100 conserved NeuN+ Hypo DMR subsets and the red lines indicate the observed values for human-derived NeuN+ Hypo DMRs. **(C) and (D)** Similar analyses for subsampling of human hyper CH DMRs to match the number and length distribution of chimpanzee hyper CH DMRs. Boxplots show the human hyper CH DMR subsets, red lines indicate observed chimpanzee hyper DMRs and blue lines indicate observed human hyper DMRs (full set). Box represents a range from the first quartile to the third quartile. The line in the box indicates the median value. The minima and maxima are within 1.5 times the distance between the first and third quartiles from box.

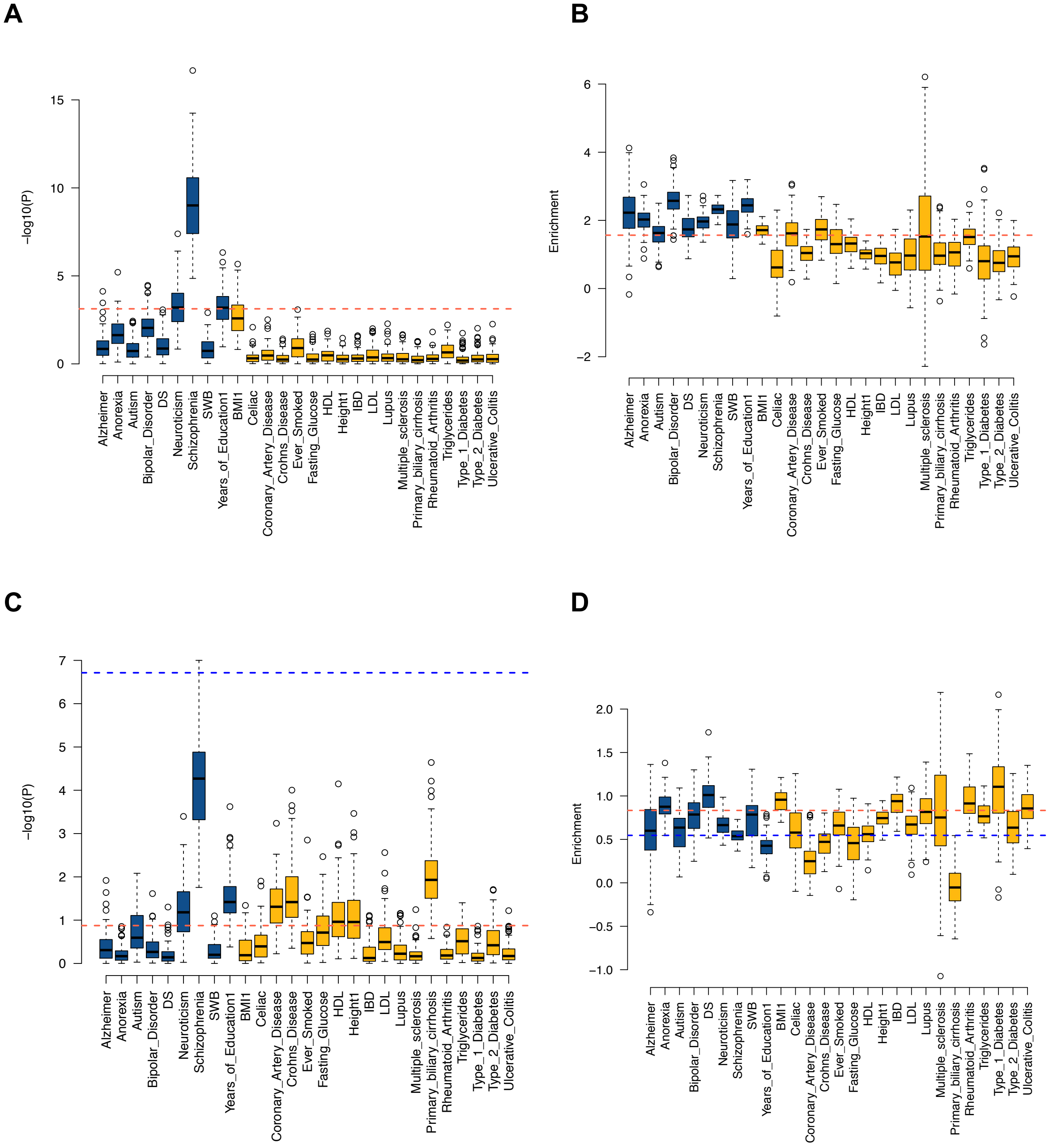

Supplementary Fig. 22. Proportions of differentially methylated loci (DML) in 5-hydroxymethylcytosines and 5-methylcytosines. We examined the cytosines that are orthologous across the three species. The proportions of DML at hmC loci (4.2%) and mC loci (4.2%) showed no difference.

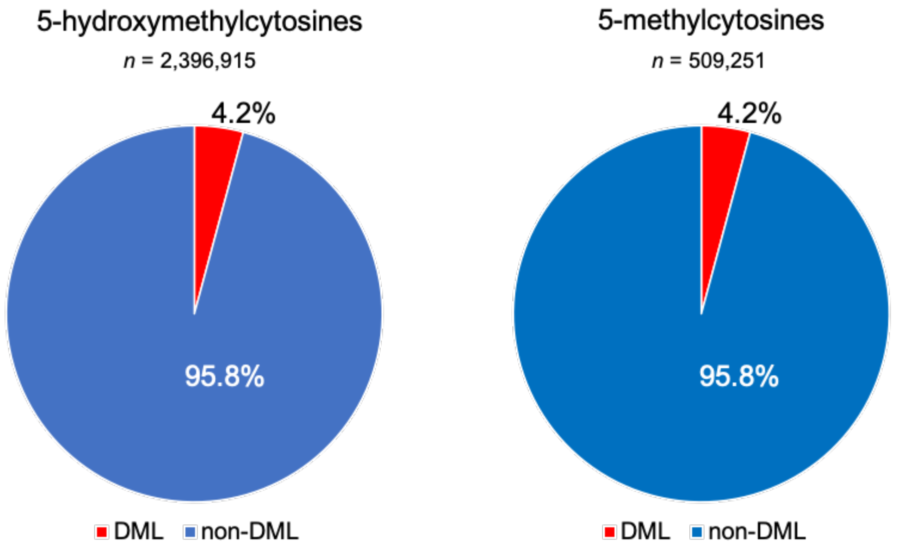

Supplementary Fig. 23. Representative FANS plot of neurons and oligodendrocytes. (A) Single nuclei were isolated by size and granularity. (B) For neuron (NeuN+) and oligodendrocyte (OLIG2+) sorting, Alexa 488 conjugated NeuN antibody and Alexa 555 conjugated OLIG2 antibody were used, respectively. “-/-” denotes neither NeuN+ nor OLIG2+ nuclei. (C) An example of nuclei population of a chimpanzee sample. Figures modified from Berto et al. 2019.

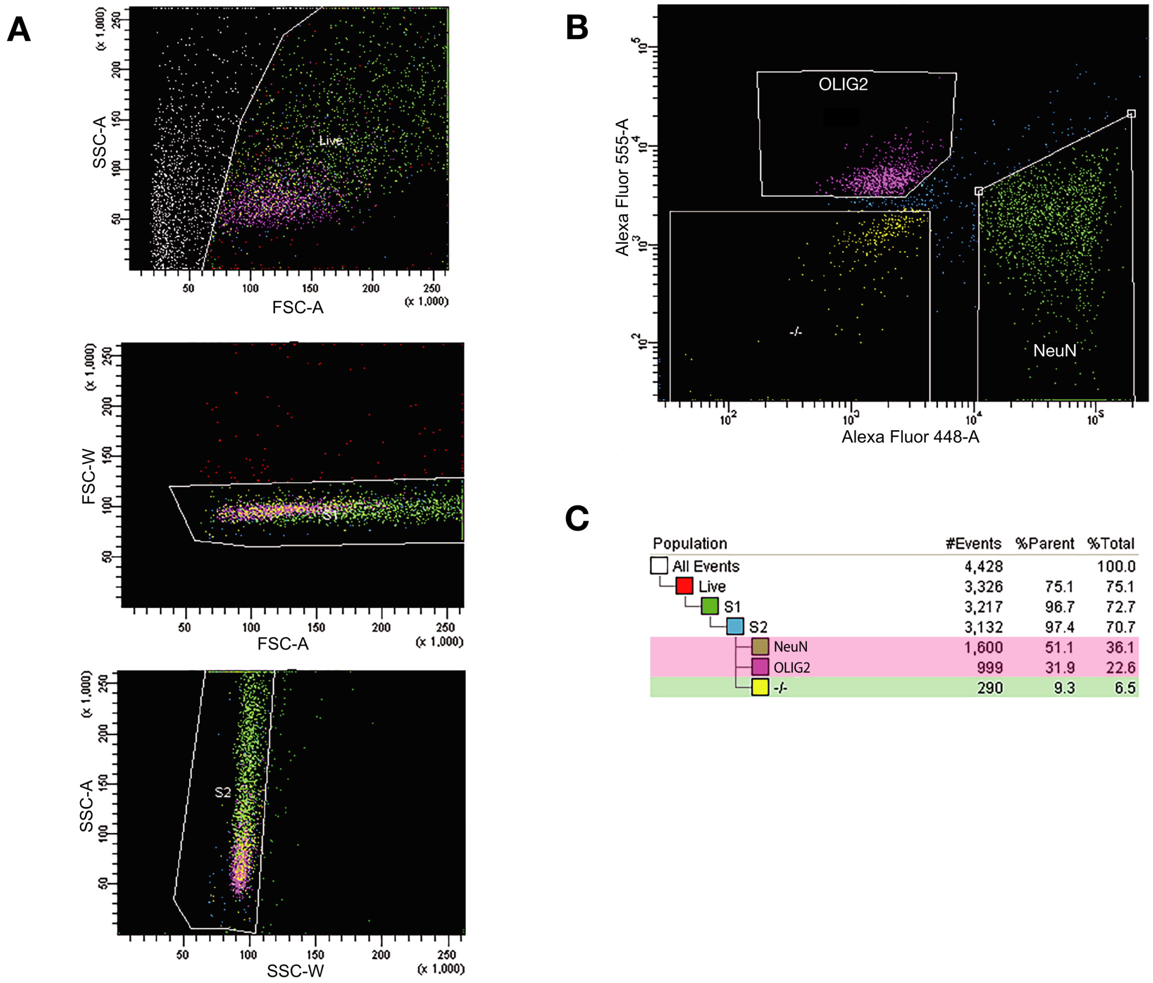

Population	#Events	%Parent	%Total
All Events	4,428		100.0
Live	3,326	75.1	75.1
S1	3,217	96.7	72.7
S2	3,132	97.4	70.7
NeuN	1,600	51.1	36.1
OLIG2	999	31.9	22.6
-/-	290	9.3	6.5

Supplementary Fig. 24. Multidimensional scaling plot (MDS) of SNPs shared among the 11 chimpanzees (whole-genome sequencing data; black diamonds) and bonobo and chimpanzees from De Manuel et al. for chromosome 20 using 75,575 common SNPs. **(A)** MDS of bonobo and chimpanzees. **(B)** MDS of chimpanzee individuals only.

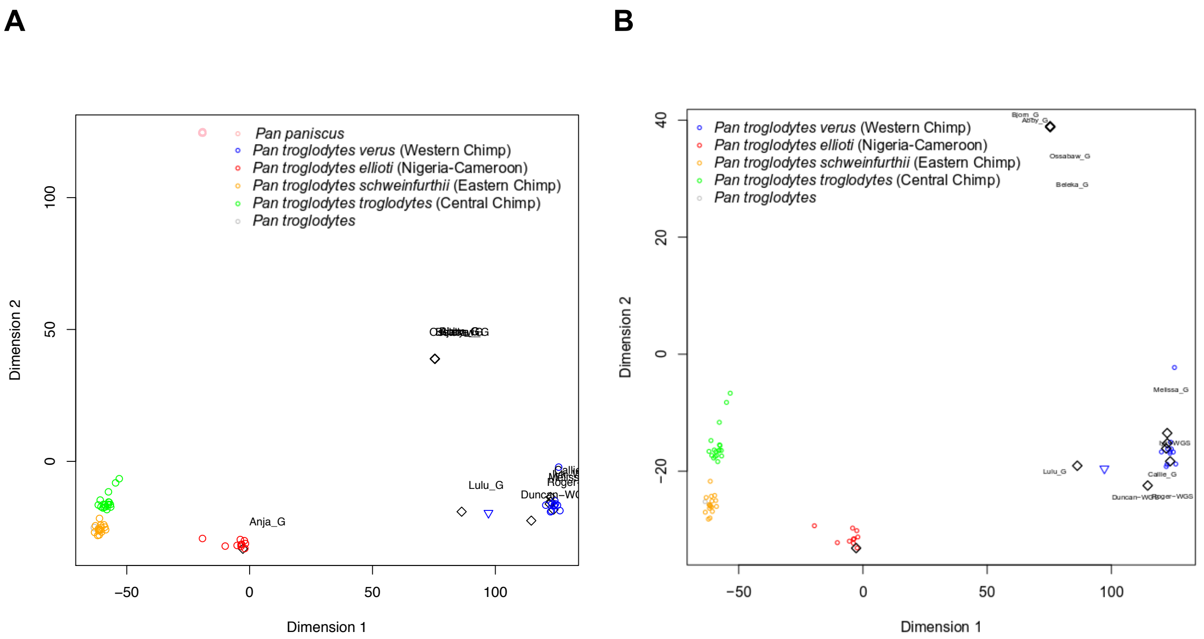

Supplement: Supplementary file 1 — Supplementary Information [file 41467_2021_21917_MOESM1_ESM.pdf]
