# Supplementary material for: Evolution of DNA methylation in the human brain
Source: Nat Commun. 2021 Apr 1;12:2021. doi: 10.1038/s41467-021-21917-7 (PMC8017017; doi:10.1038/s41467-021-21917-7)
Supplement: Supplementary file 3 — Description of Additional Supplementary Files [file 41467_2021_21917_MOESM3_ESM.pdf]

Description of Additional Supplementary Files

File Name: Supplementary Data 1
Description: List of covariates included in the WGBS analyses

File Name: Supplementary Data 2
Description: WGBS data processing and mapping statistics

File Name: Supplementary Data 3
Description: WGS data processing and mapping statistics

File Name: Supplementary Data 4
Description: Summary of SNP discovery and polymorphic CGs

File Name: Supplementary Data 5
Description: List of variable CpG-specific and conserved CpG-specific transcription factor (TF) motifs

File Name: Supplementary Data 6
Description: Genomic coordinate and annotation of CG DMRs between NeuN+ and OLIG2+ conserved in the brains of all three species

File Name: Supplementary Data 7
Description: Gene ontology (GREAT) output for genes associated with conserved CG DMRs between NeuN+ and OLIG2+

File Name: Supplementary Data 8
Description: Genomic coordinate and annotation of human- and chimpanzee- brain specific CG DMRs

File Name: Supplementary Data 9
Description: Association of differentially expressed genes (DEG) and DMR-genes

File Name: Supplementary Data 10
Description: Three-dimensional chromatin Interaction profiles for human neuron CG DMRs

File Name: Supplementary Data 11
Description: Gene ontology output (ShinyGO) for three-dimensional chromatin Interaction profiles for human neuron CG DMRs

File Name: Supplementary Data 12
Description: TF motifs significantly associated with human hypomethylated DMRs

File Name: Supplementary Data 13
Description: Partial correlation analysis explaining correlation coefficient between methylation and expression account for effects from other methylation contexts

File Name: Supplementary Data 14

Description: Multiple linear regression models explaining variation of gene expression levels of humans and human-chimpanzee difference

File Name: Supplementary Data 15
Description: Significant levels of human-specific DMR-genes for human-specific WGCNA modules

File Name: Supplementary Data 16
Description: List of CH DMR genes

File Name: Supplementary Data 17
Description: The list of GWAS traits and references used in LD score regression analyses

File Name: Supplementary Data 18
Description: LD score regression results

File Name: Supplementary Data 19
Description: List of human-specific neuron hypomethylated DMRs and conserved neuron hypomethylated DMRs harboring credible schizophrenia-associated SNPs ($P < 10^{-5}$)
