# Supplementary material for: Evolution of DNA methylation in the human brain
Source: Nat Commun. 2021 Apr 1;12:2021. doi: 10.1038/s41467-021-21917-7 (PMC8017017; doi:10.1038/s41467-021-21917-7)
Supplement: Supplementary file 5 — Reporting Summary [file 41467_2021_21917_MOESM5_ESM.pdf]

nature research

Corresponding author(s): Soojin Yi and Genevieve Konopka

Last updated by author(s): Feb 7, 2021

Reporting Summary

Nature Research wishes to improve the reproducibility of the work that we publish. This form provides structure for consistency and transparency in reporting. For further information on Nature Research policies, see our Editorial Policies and the Editorial Policy Checklist.

Statistics

For all statistical analyses, confirm that the following items are present in the figure legend, table legend, main text, or Methods section.

n/a	Confirmed	
☐	☒	The exact sample size (*n*) for each experimental group/condition, given as a discrete number and unit of measurement
☐	☒	A statement on whether measurements were taken from distinct samples or whether the same sample was measured repeatedly
☐	☒	The statistical test(s) used AND whether they are one- or two-sided<br>*Only common tests should be described solely by name; describe more complex techniques in the Methods section.*
☐	☒	A description of all covariates tested
☐	☒	A description of any assumptions or corrections, such as tests of normality and adjustment for multiple comparisons
☐	☒	A full description of the statistical parameters including central tendency (e.g. means) or other basic estimates (e.g. regression coefficient) AND variation (e.g. standard deviation) or associated estimates of uncertainty (e.g. confidence intervals)
☐	☒	For null hypothesis testing, the test statistic (e.g. *F*, *t*, *r*) with confidence intervals, effect sizes, degrees of freedom and *P* value noted<br>*Give P values as exact values whenever suitable.*
☐	☒	For Bayesian analysis, information on the choice of priors and Markov chain Monte Carlo settings
☐	☒	For hierarchical and complex designs, identification of the appropriate level for tests and full reporting of outcomes
☐	☒	Estimates of effect sizes (e.g. Cohen's *d*, Pearson's *r*), indicating how they were calculated

Our web collection on statistics for biologists contains articles on many of the points above.

Software and code

Policy information about availability of computer code

Data collection	No software was used to collect data.
Data analysis	WGS data was processed with TrimGalore v0.4.1, BWA v0.7.4, picard v2.8.3, GATK v3.7. WGBS data was processed with TrimGalore v0.4.1, Bismark v 0.14.5, bowtie v2.3.4, DSS v 2.3. For transcription factor binding motif analysis, we used the MEME suite's AME software and two HOCOMOCO v11 databases. Statistical analyses and visualizations were done using R v4.0.1.<br><br>The code for processing methylation data is available at Github at https://github.com/soojinyilab/Brain_methylome_NHP.

For manuscripts utilizing custom algorithms or software that are central to the research but not yet described in published literature, software must be made available to editors and reviewers. We strongly encourage code deposition in a community repository (e.g. GitHub). See the Nature Research guidelines for submitting code & software for further information.

Data

Policy information about availability of data

All manuscripts must include a data availability statement. This statement should provide the following information, where applicable:

- Accession codes, unique identifiers, or web links for publicly available datasets
- A list of figures that have associated raw data
- A description of any restrictions on data availability

All raw data and processed methylation data described in this study have been deposited in the NCBI Gene Expression Omnibus and available at GEO Series accession number GSE151768.

Field-specific reporting

Please select the one below that is the best fit for your research. If you are not sure, read the appropriate sections before making your selection.

- [x] Life sciences
- [ ] Behavioural & social sciences
- [ ] Ecological, evolutionary & environmental sciences

For a reference copy of the document with all sections, see nature.com/documents/nr-reporting-summary-flat.pdf

Life sciences study design

All studies must disclose on these points even when the disclosure is negative.

Sample size	No statistical methods were used to pre-determine sample sizes; adult human postmortem brain samples from Brodmann area 46 (BA46) were acquired from the National Institutes of Health NeurobioBank (the Harvard Brain Tissue Resource Center, the Human Brain and Spinal Fluid Resource Center, VA West Los Angeles Healthcare Center, and the University of Miami Brain Endowment Bank). These samples included 25 and 22 NeuN+ and OLIG2+ specimens, respectively. All non-human primate samples were obtained from homologous regions in chimpanzees (NeuN+ n = 11, OLIG2+ n = 11) and rhesus macaques (NeuN+ n = 15, OLIG2+ n = 13).<br>The numbers of NHP samples reflect the largest such data reported so far. These samples are highly limited due to their nature and no sample size calculation was performed prior to collection.
Data exclusions	No data were excluded from the analyses.
Replication	Evolutionary data cannot be easily replicated due to the uniqueness of the dataset (e.g., brain tissue samples from chimpanzees). However our data has a large number of biological replicates.
Randomization	Samples were randomized at each stage of data collection. All covariates including batch effects due to randomization were taken into account.
Blinding	Our experiments did not involve any live subjects and thus were not subject to blinding. Human data were de-identified prior to collection. NHP data collection and analyses were not performed blind to the conditions of the experiments. However, separate individuals carried out wet-bench and dry-bench analyses.

Reporting for specific materials, systems and methods

We require information from authors about some types of materials, experimental systems and methods used in many studies. Here, indicate whether each material, system or method listed is relevant to your study. If you are not sure if a list item applies to your research, read the appropriate section before selecting a response.

Materials & experimental systems

n/a	Involved in the study	
[ ]	[x]	Antibodies
[x]	[ ]	Eukaryotic cell lines
[x]	[ ]	Palaeontology and archaeology
[ ]	[x]	Animals and other organisms
[ ]	[x]	Human research participants
[x]	[ ]	Clinical data
[x]	[ ]	Dual use research of concern

Methods

n/a	Involved in the study	
[x]	[ ]	ChIP-seq
[ ]	[x]	Flow cytometry
[x]	[ ]	MRI-based neuroimaging

Antibodies

Antibodies used	mouse alexa488 conjugated anti-NeuN was used at a 1:200 dilution. Supplier = Millipore Cat #MAB377X<br>rabbit alexa 555 anti OLIG2 was used at a dilution of 1:75. Supplier = Millipore Cat #AB9610-AF555
Validation	Antibodies were used to stain nuclei and specificity was checked by microscopy. The NeuN antibody was used for similar experiments from postmortem tissue in PMID 30038276. The OLIG2 antibody is widely used for postmorterm brain experiments and iPSC experiments, including studies PMID 28246330, PMID 26776227.

Animals and other organisms

Policy information about studies involving animals; ARRIVE guidelines recommended for reporting animal research

Laboratory animals	In the supplementary data 1, we report the species, sex, and age of the samples in this study.
Wild animals	This study did not involve wild animals.

Field-collected samples	No field-collected samples are used.
Ethics oversight	For human samples, UT Southwestern Medical Center Institutional Review Board has determined that as this research was conducted using post-mortem specimens, the project does not meet the definition of human subjects research, and does not require IRB approval and oversight. Non-human primate samples were obtained following all relevant ethical regulations and institutional review board of the Emory Institutional Animal Care and Use Committee.

Note that full information on the approval of the study protocol must also be provided in the manuscript.

Human research participants

Policy information about studies involving human research participants

Population characteristics	*Describe the covariate-relevant population characteristics of the human research participants (e.g. age, gender, genotypic information, past and current diagnosis and treatment categories). If you filled out the behavioural & social sciences study design questions and have nothing to add here, write "See above."*
Recruitment	*Describe how participants were recruited. Outline any potential self-selection bias or other biases that may be present and how these are likely to impact results.*
Ethics oversight	*Identify the organization(s) that approved the study protocol.*

Note that full information on the approval of the study protocol must also be provided in the manuscript.

Flow Cytometry

Plots

Confirm that:

- [x] The axis labels state the marker and fluorochrome used (e.g. CD4-FITC).
- [x] The axis scales are clearly visible. Include numbers along axes only for bottom left plot of group (a 'group' is an analysis of identical markers).
- [x] All plots are contour plots with outliers or pseudocolor plots.
- [x] A numerical value for number of cells or percentage (with statistics) is provided.

Methodology

Sample preparation	Nuclei were extracted from approximately 700mg of frozen human cortical tissue. The nuclei were incubated with antibodies for NeuN and OLIG2 and then sorted using flow cytometry. Isolated nuclei were then processed to obtain gDNA and nuclear RNA.
Instrument	FACS Aria IIU; BD Biosciences
Software	BD FACS Diva Software
Cell population abundance	The relevant information is reported in Berto et al. 2019 PNAS where transcriptome data were generated.
Gating strategy	Nuclei were first sorted for size and complexity, followed by gating to exclude doublets that indicate aggregates of nuclei. The nuclei were then further sorted based on fluorescence: alexa 488 for NeuN positive populations and alexa 555 for OLIG2 positive populations. A figure of the gating strategy is now provided as a new Supplementary Figure 23.

- [x] Tick this box to confirm that a figure exemplifying the gating strategy is provided in the Supplementary Information.
